# Draft Genome Sequence of Streptomyces thermocarboxydus BPSAC147, a Potentially Plant Growth-Promoting Endophytic Bacterium

**DOI:** 10.1128/MRA.00363-19

**Published:** 2019-06-06

**Authors:** Ajit Kumar Passari, Vinay Rajput, Zothanpuia, Lakshmi P. M. Priya, Mahesh Dharne, Syed Dastager, Oommen K. Mathew, Abeer Hashem, Elsayed Fathi Abd_Allah, Bhim Pratap Singh

**Affiliations:** a Department of Biotechnology, Mizoram University, Aizawl, Mizoram, India; b NCIM Resource Centre, CSIR-National Chemical Laboratory, Pune, India; c AgriGenome Labs Pvt Ltd., Kochi, India; d Botany and Microbiology Department, College of Science, King Saud University, Riyadh, Saudi Arabia; e Mycology and Plant Disease Survey Department, Plant Pathology Research Institute, Agriculture Research Center, Giza, Egypt; f Plant Production Department, College of Food and Agriculture Science, King Saud University, Riyadh, Saudi Arabia; g Departamento de Biologia Molecular y Biotecnologia, Instituto de Investigaciones Biomedicas, Universidad Nacional Autonoma de Mexico (UNAM), Mexico City, Mexico; Indiana University, Bloomington

## Abstract

Streptomyces thermocarboxydus strain BPSAC147 is an endophytic actinobacterium isolated from root tissues of Rhynchotechum ellipticum in Mizoram, Northeast India. The strain showed potentially plant growth-promoting and biocontrol activities. The draft genome consists of 7.37 Mb and encodes 7,060 putative open reading frames.

## Abstract (precis)

Streptomyces thermocarboxydus strain BPSAC147 is an endophytic actinobacterium isolated from root tissues of Rhynchotechum ellipticum in Mizoram, Northeast India. The strain showed potentially plant growth-promoting and biocontrol activities.

## ANNOUNCEMENT

Plant growth-promoting endophytic microorganisms are present inside plant tissues in either symbiotic or mutualistic association with their host plant without exhibiting negative effects on the host plant or the environment (1). Streptomyces thermocarboxydus BPSAC147 was isolated from root tissues of Rhynchotechum ellipticum cultivated in Mizoram, India. After surface sterilization, root fragments were placed onto petri plates containing tap water-yeast extract agar (TWYE) medium supplemented with nystatin and cycloheximide (60 μg/ml) to suppress fungal growth. Then, the plate was incubated at 28°C for 15 days (2). Based on 16S rRNA sequence analysis, strain BPSAC147 is closely matched to Streptomyces thermocarboxydus strain DSM44293, with 99.8% similarity (GenBank accession number KP128880). A Streptomyces thermocarboxydus isolate showed the ability to solubilize phosphate and produce indole-3-acetic acid (IAA), ammonia, cellulase, and amylase enzyme (2). Moreover, strain BPSAC147 has a potential role as a biocontrol agent against three fungal phytopathogens, i.e., Fusarium proliferatum, Fusarium oxy f. sp. ciceri, and Fusarium oxysporum (2). Here, we present the draft genome sequence of Streptomyces thermocarboxydus BPSAC147.

A single colony was transferred to 10 ml of tryptone broth (ISP1 broth) and incubated at 28°C for 7 days at 150 rpm. Genomic DNA was extracted using a Purelink genomic DNA isolation kit (catalog number K182002; Thermo Scientific Invitrogen), and 500 ng of good-quality genomic DNA was fragmented using an M220 instrument (Covaris, Inc.). Fragmented genomic DNA was end repaired and further processed for ligation of Illumina adaptors with a NEBNext Ultra DNA library preparation kit (catalog number E7370L; New England BioLabs [NEB]), per the recommendation of the manufacturer. The adaptor-ligated enriched library was purified using AMPure XP beads. Library size distribution was checked on an Agilent Tapestation D1000 DNA chip (catalog number 5067-5583). The Streptomyces thermocarboxydus BPSAC147 genomic DNA was sequenced with the 2 × 150-bp paired-end read length sequencing protocol of the Illumina MiSeq platform. The quality check of the reads was done using FastQC (3), and the generated sequencing reads were filtered to remove low-quality reads using Trim Galore v0.5.0 (4) with set default parameters. Unicycler v0.4.8 (5) was used for *de novo* assembly with an *N*_50_ value of 503,505 bp. The Streptomyces thermocarboxydus BPSAC147 draft genome sequence contains 103 contigs and an estimated genome length of 7,375,534 bp, with an average G+C content of 72.01%.

The Streptomyces thermocarboxydus strain BPSAC147 genome was annotated on the PATRIC Web server (6) using the Rapid Annotations using Subsystems Technology tool kit (RASTtk) (7), which contains 7,060 protein-coding sequences (CDS), 66 tRNA genes, and 4 rRNA genes. This genome was annotated using genetic code 11, and the taxonomy of this genome is cellular organisms > bacteria > *Terrabacteria* group > *Actinobacteria* > *Streptomycetales* > *Streptomycetaceae* > *Streptomyces* > Streptomyces thermocarboxydus. The annotation of Streptomyces thermocarboxydus strain BPSAC147 includes 2,506 hypothetical proteins and 5,053 proteins with functional assignments. The proteins with functional assignments included 1,235 proteins with Enzyme Commission (EC) numbers, 1,072 with Gene Ontology (GO) assignments, and 969 proteins that were mapped to KEGG pathway.

### Data availability.

This whole-genome shotgun project has been deposited at DDBJ/EMBL/GenBank under accession numbers STGO01000001 to STGO01000102. The version described in this paper is the first version, STGO01000000. The Sequence Read Archive accession number is SRS4499771, and the BioProject accession number is PRJNA527710.
